# Visualizing tactile feedback: an overview of current technologies with a focus on ultrasound elastography

**DOI:** 10.3389/fmedt.2023.1238129

**Published:** 2023-10-03

**Authors:** Avisha Kumar, Kelley M. Kempski Leadingham, Max J. Kerensky, Sriramana Sankar, Nitish V. Thakor, Amir Manbachi

**Affiliations:** ^^1^^ Department of Electrical and Computer Engineering, Johns Hopkins University, Baltimore, MD, United States; ^^2^^ HEPIUS Innovation Lab, Johns Hopkins University School of Medicine, Baltimore, MD, United States; ^^3^^ Department of Neurosurgery, Johns Hopkins University School of Medicine, Baltimore, MD, United States; ^^4^^ Department of Biomedical Engineering, Johns Hopkins University School of Medicine, Baltimore, MD, United States; ^^5^^ Department of Mechanical Engineering, Johns Hopkins University, Baltimore, MD, United States; ^^6^^ Department of Anesthesiology and Critical Care Medicine, Johns Hopkins University School of Medicine, Baltimore, MD, United States

**Keywords:** ultrasound imaging, elastography, tactile sensors, tissue palpation, micromachined ultrasound transducers

## Abstract

Tissue elasticity remains an essential biomarker of health and is indicative of irregularities such as tumors or infection. The timely detection of such abnormalities is crucial for the prevention of disease progression and complications that arise from late-stage illnesses. However, at both the bedside and the operating table, there is a distinct lack of tactile feedback for deep-seated tissue. As surgical techniques advance toward remote or minimally invasive options to reduce infection risk and hasten healing time, surgeons lose the ability to manually palpate tissue. Furthermore, palpation of deep structures results in decreased accuracy, with the additional barrier of needing years of experience for adequate confidence of diagnoses. This review delves into the current modalities used to fulfill the clinical need of quantifying physical touch. It covers research efforts involving tactile sensing for remote or minimally invasive surgeries, as well as the potential of ultrasound elastography to further this field with non-invasive real-time imaging of the organ’s biomechanical properties. Elastography monitors tissue response to acoustic or mechanical energy and reconstructs an image representative of the elastic profile in the region of interest. This intuitive visualization of tissue elasticity surpasses the tactile information provided by sensors currently used to augment or supplement manual palpation. Focusing on common ultrasound elastography modalities, we evaluate various sensing mechanisms used for measuring tactile information and describe their emerging use in clinical settings where palpation is insufficient or restricted. With the ongoing advancements in ultrasound technology, particularly the emergence of micromachined ultrasound transducers, these devices hold great potential in facilitating early detection of tissue abnormalities and providing an objective measure of patient health.

## 1. Introduction

Many diseases and health concerns have a shorter treatment time and a higher rate of survival if detected at an early stage. During annual checkups, physicians traditionally use manual palpation to assess the progression of illnesses, either by taking arterial (e.g., carotid, radial) pulse measurements or by detecting tumor nodules in soft tissue (e.g., lymph nodes, breast masses) (1). Because the tissue’s elasticity is variable depending on its health, this practice involves applying pressure to tissue or organs (1–5 cm depression) and using tactile feedback to localize stiff nodules or irregularities that need to be examined further (2). However, the physician’s reading of patient health is limited by tissue depth, practitioner experience, and thick layers of fat or ascites covering the region of interest, raising the need for noninvasive and quantitative appraisals of tissue elasticity.

Furthermore, while physicians can rely on their sense of touch to qualitatively assess the biomechanical properties of the tissue at the bedside, there is a clear lack of tactile feedback in minimally invasive and remote surgeries. Although these procedures have become increasingly popular due to expedited healing times and mitigated risks for infection, pain, blood loss, and trauma, surgeons lose their ability to palpate tissue (3). Tactile feedback is crucial for interoperative decisions, like determining locations for incisions, and patient safety from surgical instruments. There have been various efforts to develop sensors to assess tissue stiffness in these settings to recoup physical touch (4). These systems must enable a reliable and quantifiable method to “palpate” tissue by not only capturing the response to pressure but also by measuring the applied force to accurately interpret the feedback. There are 4 main types of sensors for tactile feedback: resistive, capacitive, piezoelectric, and optical (4). These sensors have made tremendous strides in the field of minimally invasive surgeries, allowing clinicians to assess tissue characteristics in cases where manual palpation is impractical due to small incisions. However, they solely provide sensory information and do not offer noninvasive imaging of the tissue’s elastic profile.

Ultrasound can fulfill this visual shortcoming by enabling real-time, noninvasive imaging. It is a widely accepted clinical tool that emits sound waves at high frequencies (≥20 kHz) to treat and image tissues without ionizing radiation. Therapeutic ultrasound focuses high-intensity acoustic waves to a precise location to remotely treat the tissue, either by modifying it (e.g., muscle therapy, neuromodulation) with intensities at 1–4 W/cm${}^{2}$, or damaging it (e.g., tumor ablation, dissolving blood clots) using intensities greater than 1 kW/${\text{cm}}^{2}$ (5, 6). Diagnostic ultrasound, on the other hand, provides imaging capabilities at much lower intensities so that the tissue can be assessed for any abnormalities. These transducers emit ultrasound waves and receive the echoed signal that reflects off any structures it encounters (7, 8). Studies have shown that diagnostic ultrasound yields more consistent and accurate diagnoses compared to traditional palpation methods, with a 10-fold increase in accuracy when using ultrasound in some applications (9, 10). This can largely be attributed to the visual capabilities of ultrasound, where stiffness signals are quantitatively processed and displayed to the practitioner, unlike with traditional palpation where the clinician can only rely on qualitative metrics like physical touch. Diagnostic ultrasound is safer, less expensive, and more portable than radiography, computed tomography (CT), and magnetic resonance imaging (MRI); these benefits allow for easier integration of ultrasound into robotic and teleoperative systems for remote diagnosis (11). In this review, we focus on diagnostic ultrasound—specifically, elastography—for its ability to augment tactile feedback. Ultrasound elastography can mimic manual palpation in both superficial and deep structures in a quantifiable manner, providing clinically valuable insights.

The next section of this review describes the sensors used for visualizing tactile feedback in clinical settings. Subsequently, we delve into ultrasound elastography and the 2 main modalities used to measure tissue elasticity: (1) strain and (2) shear wave imaging. We focus on the fundamentals of these imaging techniques and explore the potential growth of this practice using advancements in ultrasound probe fabrication (i.e., micro-electromechanical systems). Finally, the integration of elasticity imaging into clinical ultrasound devices for visualizing tactile feedback is discussed. The paper concludes with a consideration of the limitations of these systems as well a discussion on the increased accessibility and scope of elastography due to breakthroughs in artificial intelligence and wearable technology.

## 2. Sensors for tactile feedback

Since tissue responses vary based on the input force and tissue health, tactile sensors that quantify the applied pressure and feedback can serve as an alternative to manual palpation. Moreover, compared to open surgeries, where physicians can rely on their sense of touch and vision to localize tumors, blood vessels, and tissue swelling, it is significantly more challenging for physicians to receive tactile feedback in minimally invasive surgeries. While MRI or CT scans can provide stiffness information of the anatomy before surgery, there are several causes of mismatch between preoperative and interoperative patient status, including organ shift, swelling, and deformations.

**Figure 1:**
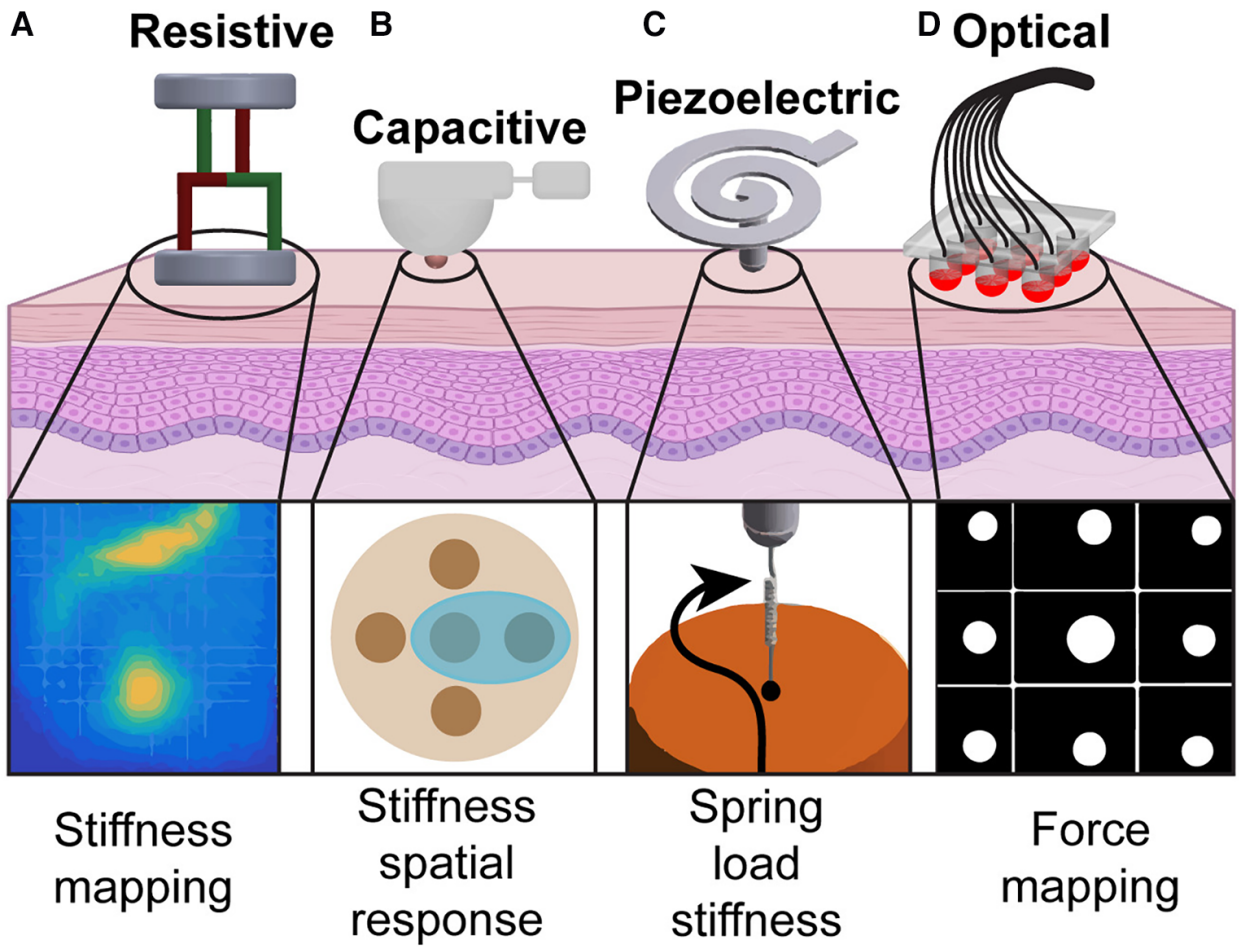
Tactile sensing modalities used in tissue palpation: (**A**) By probing the organ at several points, a stiffness map of the underlying tissue is created using resistive sensors (13). (**B**) A palpation probe with a capacitive sensor consisting of multiple sensing elements is used to analyze stiffness variation during palpation (14). (**C**) Vibration generated by the probe tip on the tissue surface creates a spring-based mechanical impedance that is measured by the piezoelectric sensor to identify the spatial distribution of tissue hardness (15). (**D**) Probe head using fiber optic cables is used to palpate tissues and generate a force map to identify nodules (16).

There are several factors to consider when designing sensors for surgical and diagnostic applications, including resolution, size, weight, sensitivity, biocompatibility, sterilizability, and modularity (4). The 4 main types of tactile sensors used for supplementing tissue palpation are resistive, capacitive, piezoelectric, and optical sensors (Figure 1) (12). These sensors obtain information through physical touch and can characterize tumor properties such as size, depth, and elasticity through hardness or pressure measurements. The primary differences between these sensors are their transduction methods. While there are several reviews that focus on the fabrication methods, physical theories, and biomedical applications of these sensors (17, 18, 12), this section aims to highlight the sensing devices that are specifically important for visualizing tactile feedback in clinical settings. Table 1 provides an overview of these technologies and their applications.

**Table 1:** Sensors for tactile feedback.

Sensor	Operating principle	Application	Reference
Resistive	Resistance changes in metal strain gauges result from changes in geometry (e.g., length, cross-section)	Stiffness mapping	(13, 19)
		Hard nodule detection	
		Robotic tissue palpation	
Capacitive	Two conductive objects with a space between them respond to applied voltage differences	Probe that mimics human fingertip tactile sensing	(14, 20)
		Surgical forceps for tactile feedback	
Piezoelectric	Applied mechanical stress can generate an electric response	Robotic-based tissue palpation system	(15, 21, 22)
		Portable pen-like devices for oral cancer screening	
		Measuring elasticity of tissue	
Optical	Acts as a wavelength-selective mirror in which light within a narrow spectral width will be reflected and remaining light will be transmitted	Fiber Bragg Grating-based force sensors used in robotic palpation for haptic perception in surgeries	(16)

### 2.1. Resistive sensors

A resistive sensor is a sensor that converts an application of an external force to a change in electrical resistance, allowing for force, temperature, pressure, or displacement measurements with high resolution. Strain gauges, which are a type of resistive sensor, are leveraged in robotic designs for minimally invasive surgeries for measuring tissue stiffness. With the working principle that resistance is directly proportional to the length and inversely proportional to the area of the conductor, deformation to the conductor’s geometry from an applied force can be measured. Low-cost triaxial force sensors have been developed for hard nodule detection using an electronic system of force-sensitive resistors (13). This system produces stiffness maps representing the underlying tissue by probing the surface of the organ at several points following a “raster-scan” pattern and measuring the magnitude of the force (Figure 1A).

Beccani et al. (19) introduced a interoperative wireless palpation probe (15 mm diameter, 60 mm length) that can be deployed through small incision sites to measure indentation pressure, indentation depth, and probe position during surgery (19). This probe can be directly controlled by the surgeon and provides real-time volumetric stiffness distribution maps to assist with tumor localization and interventional decisions (19). The local tissue stiffness is estimated by considering both the probe’s indentation depth during operation and the tissue reaction pressure, which is measured by a barometric pressure sensor. This approach demonstrates a stiffness error of less than 8%. Additionally, a soft robotic skin for tissue stiffness mapping was developed using soft robotic tactile elements that individually record and imitate tissue response to compression using a barometric pressure sensor (23). This robotic skin can be deployed from the incision site and creates the stiffness map as it expands into the tissue of interest (up to 3.25 mm deep). Each tactile element is equipped with a pressure sensor, a fluidic chamber that expands when introduced to an incompressible liquid, and a suction gripper so the sensor can maintain contact with the tissue and compensate for expansion forces.

It is important to note that resistive sensors come with several challenges that need to be addressed for reliable clinical use. These challenges include lower sensitivity compared to other modalities and high power consumption. Moreover, the hysteresis characteristic of resistive sensors, which results in a difference in output of any measurement value upon a change in direction (e.g., approaching the measurement value with increasing pressure first and then with decreasing pressure), may result in lack of reliability and repeatability unless compensated with nonlinear calibrations or sensor design (24).

### 2.2. Capacitive sensors

Capacitive sensing is often favored in medical device design due to its high electrical sensitivity, low power consumption, and repeatability (12). In principle, capacitive sensors have 2 conductive plates separated by a gap. The electric field between the plates changes when a conductive object comes near the sensor, and this resulting change in capacitance is detected and measured. Capacitive sensors can be densely fabricated due to their small size, achieving high resolution (approximately 1 mm) comparable to human mechanoreceptors (18).

One particular advancement in robotic probes for soft tissue palpation includes a variable lever mechanism that allows for stiffness control to enhance tumor detection accuracy and improve localization and depth estimation of abdominal organs (14). This probe consists of a capacitive tactile sensor that mimics cutaneous perception of the human fingertip, covering an area of 780 mm${}^{2}$ with 20 sensing elements. A torque sensor at the base of the probe imitates kinesthetic force feedback, providing a second sensing modality to further examine probe soft tissue during palpation and detect uncharacteristically stiff inclusions (Figure 1B). Additionally, surgical forceps have been developed with force and torque sensing capabilities using 3-degrees-of-freedom capacitive-based force sensors (20). The sensors, which are integrated into each jaw of the forceps, are designed to measure shear and normal forces allowing for applied force recordings from the instrument while grasping, manipulating, and pressing during surgery to minimize tissue damage.

Despite the many advantages of capacitive sensors, it is important to note their limitations. These sensors are prone to exhibit high non-linearity, causing discrepancies between the output signal and the measured applied force especially at high sensitivities. This requires additional signal processing to ensure the output signal is representative of the measured force. Additionally, these sensors are highly sensitive to changes in humidity and temperature, susceptible to noise and limited in sensing range, which restrict their applications (25). Moreover, capacitive sensing systems to visualize tissue stiffness maps are imprecise, costly, and require extensive calibration, which limit commercialization opportunities in clinical markets.

### 2.3. Piezoelectric sensors

Piezoelectric sensors, typically made with ceramic or crystal materials, generate electrical charge proportional to the applied physical force (e.g., pressure, temperature, vibration) (12). Several sensing devices for restoring tactile feedback either do not meet the size constraints of minimally invasive surgical robots (<12.7 mm in diameter) or solely provide the interaction force during surgery rather than a quantification of tissue hardness (15). Ju et al. (15) developed a robot-based sensor using a piezoelectric transducer attached to a spiral-shaped tactile sensor (less than 8 mm in diameter) to address the uncompromising size restrictions in minimally invasive procedures (Figure 1C) (15). This device measures the hardness (0–1.7-MPa range) of the tissue with high sensitivity by coupling the electrical impedance of the sensor and the mechanical impedance of the load. This is achieved with their unique sensor design, which consists of a spiral metal cantilever beam, a piezoelectric biomorph (two active piezoelectric layers adhered together), and a probe with a glass tip. The relationship between the electrical impedance of the sensor and the mechanical impedance of the load is derived with a piezoelectric-coupled transducer equation described in (15). The spiral shape of this system maximizes its interaction with biological tissue for hard tissue sensing while maintaining a low operating frequency.

Similarly, portable pen-like devices with miniaturized tactile sensors were made to quantitatively measure tissue elasticity for oral cancer screening (21). This probe utilizes a piezoelectric sensing film that translates the tissue’s response to pressure and detects elastomers with stiffness values ranging from 0.2 to 3.1 MPa. The sensing mechanism consists of a 2-spring model, with a hard copper ball encompassed by a soft elastomeric polymer. This creates a non-uniform stress distribution on the piezoelectric sensing film due to the varying stiffness between the ball and the polymer. As this probe presses against soft tissue, there is greater deformation in the surrounding polymer compared to the region with the ball because the polymer can conform to the depressed tissue. In comparison, when the sensor contacts hard tissue, the surrounding polymer region undergoes minimal shape distortion since the tissue is inherently less elastic and experiences reduced deformation. This variation is measured in the piezoelectric film to quantify tissue stiffness.

To optimize the performance of tactile sensors in real-time, variable-impedance piezoelectric-based sensors can measure tissue hardness with a 6.2 times increase in measurement range. The sensing range is evaluated against a set threshold signal-to-noise ratio for effective measurement. This sensing mechanism varies the mechanical impedance of the tactile element to improve sensitivities at various stiffness levels by using a unique double-cantilever structure (22). This design consists of 2 main components: an active piezoelectric bimorph and a passive length-adjustable steel wire probe. To tune the sensitivity and sensing range, the mechanical impedance can be varied by adjusting the length of the passive cantilever.

Similar to capacitive sensors, piezoelectric sensors are also temperature sensitive—a challenging limitation in a surgical set up where temperature varies up to 20${}^{\circ }$C between the operating room and core-body (26). Furthermore, piezoelectric sensors cannot detect static loads, which is crucial for surgical applications (3). Because these sensors are typically designed for detecting dynamic mechanical forces, static or slowly changing loads lead to inaccurate measurements. When clinicians need tactile feedback to inform surgical decisions, the instruments generally exert steady or slowly changing loads. Here, accurate force feedback information is critical for surgical navigation and prevention of tissue damage, and the lack of sensitivity to low forces and poor spatial resolution poses a major issue.

### 2.4. Optical sensors

Optical sensors convert changes in the properties of light (e.g., polarization, intensity, wavelength) into electronic signals. These sensors exhibit high accuracy, resolution, bandwidth, and immunity to electromagnetic interference, which is ideal for system integration (27). One of the most common types of optical tactile sensors are fiber Bragg grating (FBG) sensors. In these sensors, the index of refraction within the core of the optical fiber is modulated along its length so that it only reflects certain wavelengths of light, depending on the refractive index and the space between gratings. Applied forces (e.g., stretching, compression) to the FBG sensor can affect both the refractive index and grating interval, resulting in a wavelength shift of the outputted light. This shift is measured to quantify the applied force (28). High-precision and miniaturized FBG-based force sensors measure the force feedback from tissue displacement to provide surgeons with haptic perception. An FBG-based tactile force sensor, consisting of a miniature force-sensitive elastic element and a suspended optical fiber integrated with an FBG element, provides effective tumor detection (29). This sensor uses a rigid suspension configuration for improved sensitivity and resolution.

Furthermore, FBG-based 3-axis tactile sensors have been proposed for a more comprehensive haptic perception tool in surgeries (Figure 1D) (16). Five optical fibers merged with FBG sensors are suspended in a deformable medium and measure the compression or tension of the tissue as the sensors are pressed against it, returning a surface reaction map. While FBG-based sensors are small, flexible, and sensitive, there are several challenges that need to be addressed for optimal performance for tactile feedback. These sensors are temperature sensitive, requiring temperature compensation for accurate measurements (3). Additionally, optical sensors have short-lived stability, requiring advanced fabrication and signal processing for clinical applications (27).

Despite many research efforts to integrate tactile sensing in minimally invasive surgeries, to our knowledge there are no established mainstream commercialized products. Regardless, it is evident that these sensors provide a quantitative alternative to manual palpation, especially when clinicians have limited sense of touch in robotic-assisted surgeries (4). Although these sensors provide many advantages for tactile feedback restoration or augmentation, a noninvasive real-time imaging modality such as ultrasound offers several additional benefits. Furthermore, ultrasound elastography overcomes the limitations commonly associated with many of these sensing modalities, such as temperature-sensitivity, sensing range, and data visualization. The following sections of this review will focus on ultrasound imaging and elastography for visualizing tactile feedback.

## 3. Ultrasound for elasticity imaging

Ultrasound makes up approximately a third of the global medical imaging market (30), and is projected to be worth 11.6 billion USD by 2028. Ultrasound elastography, specifically, has been widely adopted for clinical use in liver (31), breast (32), musculoskeletal (33), and thyroid (34) applications with potential to address unmet needs in minimally invasive surgeries (35) and lung disease assessment (36). Elastography maps the stiffness in soft tissue noninvasively, providing diagnostic information to detect or monitor disease. It is especially useful in cases where the dermis and subcutaneous fat layer inhibit accurate tissue characterization with manual palpation (37). While ultrasound elastography is computationally expensive compared to the sensing modalities discussed in section 2, it offers competitive benefits for visualizing tactile feedback. With an increased depth of sensing (up to 10 cm), noninvasive real-time imaging of the anatomy, portability, ease of operation, and intuitive display of stiffness, elastography improves performance and reliability for clinical applications (38). Here, we discuss the modalities of ultrasound elastography that are relevant for visualizing tactile feedback.

**Figure 2:**
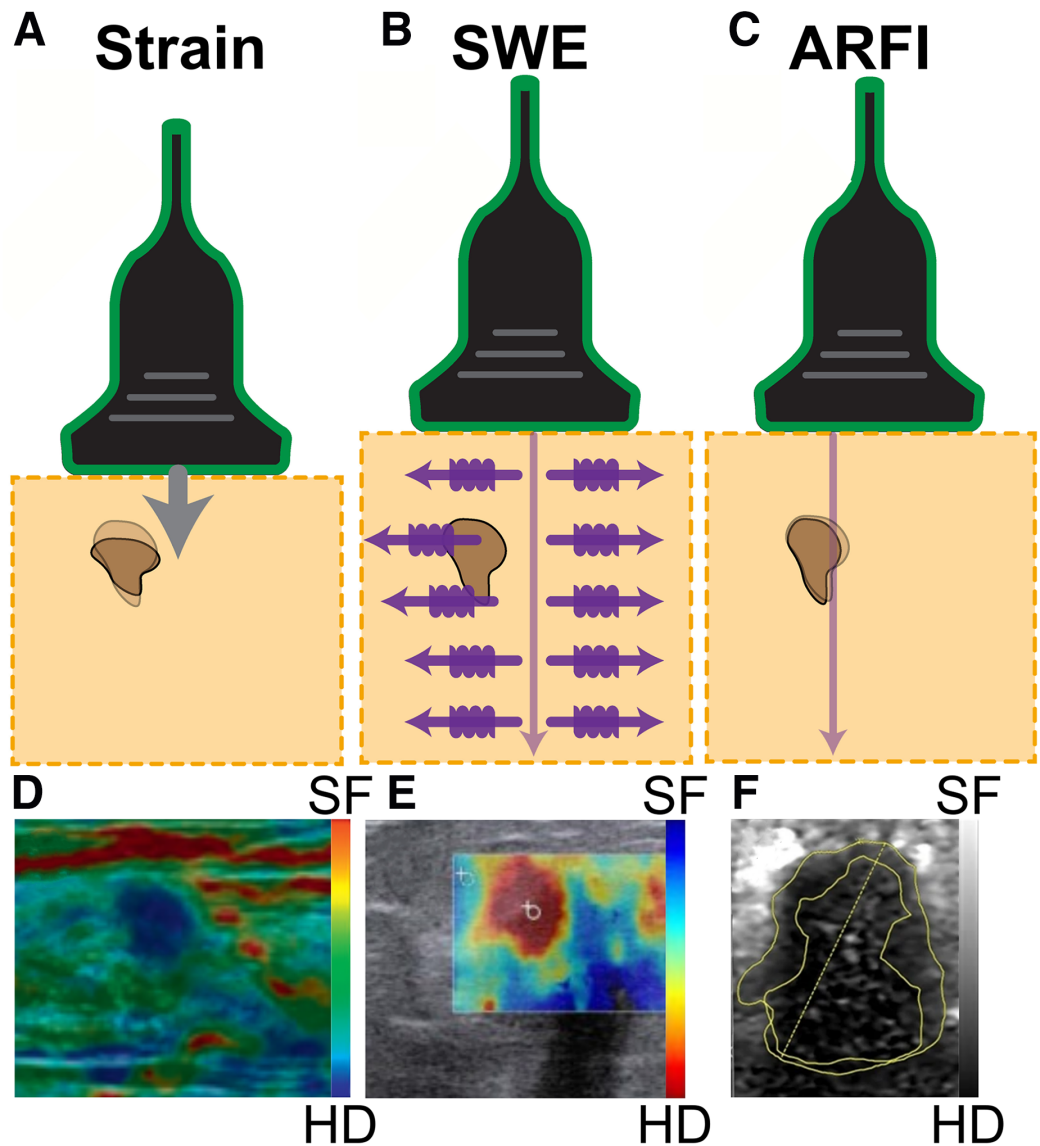
Three common ultrasound elastography modalities. (**A**) Strain elastography applies a physical compression force from the transducer, computing stiffness based on the resulting tissue displacements. This technique provides qualitative information on the relative stiffness between tissues, where tissues exhibiting greater resistance to the applied manual compression indicate higher stiffness. The hard nodule, indicated in dark brown, is compressed from the physical pressing of the transducer, which is represented by the arrow. The shadow of the dark mass indicates the original form of the nodule, and the fully colored form is the present form of the mass due to physical pressure being applied to it. (**B**) Shear wave elastography (SWE) emits a pulse wave into the tissue and tracks the perpendicularly-induced propagating shear waves. The tracked speed is computed into an elasticity value as waves travel faster through stiffer tissues including the brown lump in the figure. (**C**) Acoustic radiation force impulse (ARFI) imaging tracks the impulse response of the tissue after generating displacement at the region of interest with an ultrasound pulse, shown with the arrow. The change in shape of the hard nodule, indicated by the brown mass, is due to the application of ultrasound pressure. Detection of a breast mass is captured by (**D**) strain elastography, (**E**) shear wave elastography (SWE), and (**F**) acoustic radiation force impulse (ARFI). In each image, the breast mass was hard (HD), while the surrounding tissue was soft (SF). Images were adapted from (39, 38, 41), respectively.

The two main techniques for elastography are strain imaging and shear wave imaging (39, 40). Strain imaging is based on the principle of applying a normal stress (e.g., pressure or force) on the tissue and measuring the corresponding strain (i.e., tissue deformation) (Figure 2A). It provides qualitative information on tissue stiffness. On the other hand, shear wave imaging quantitatively measures the resulting shear waves speed from an applied force perpendicular to the tissue. The ultrasound transducer senses the speed of the vibrations from the perpendicularly-induced propagating shear waves and computes the corresponding elasticity value. In Figure 2B, we show shear waves induced by acoustic radiation force (ARF) propagating perpendicular to the ultrasound beam to detect (and image) varying degrees of tissue stiffness. ARF is a phenomenon that occurs when sound waves exert a mechanical force on an object, causing it to experience displacement or deformation. While ARF includes the word *radiation* in the name, it is important to note that it involves no ionizing radiation and the method remains safe for patients and clinical operators. Measuring the velocity and displacement of these shear waves provides insight into the tissue properties with faster waves indicating stiffer tissue. These modalities and the resulting clinical tools and applications will be discussed in the following subsections.

### 3.1. Strain imaging

Strain imaging can be broadly classified into 2 main categories by the measured physical quantity: strain elastography and acoustic radiation force impulse (ARFI) strain imaging (38). For strain elastography, 2 excitation methods can be used to apply the stress. The first excitation method involves applied manual compression, where the operator can exert pressure on the tissue with the ultrasound transducer to image superficial organs. The other excitation method requires the transducer to be held stationary while the internal physiological mechanisms (e.g., cardiovascular movement, respiration) induce the tissue deformation that is measured (42). This method is better suited to assess deeper internal organs as it is not dependent on the applied pressure of the operator. Both excitation methods provide qualitative measurements of tissue elasticity, and the strain measurements are overlaid on top of a B-mode ultrasound image as a color map (elastogram), shown in Figure 2D (39).

Although strain elastography enables tissue elasticity visualization, it can be challenging to reproduce. Since the measurements from this method use external stimuli, the resulting assessment is subjective to the manual pressure administered by the operator and the contact angle of the transducer. Additionally, this modality does not inherently correct for internal sources of stress introduced by respiratory and cardiac processes. In order to completely quantify this process, an applied force sensor or automatic indentation system must be implemented. Finally, commercial ultrasound elastography systems rely on assumptions of the tissue, such as linearity, elasticity, incompressibility, and symmetry (38). While these assumed characteristics provide a suitable initial approximation, it is important to note that these measurements can improve with analyses that more accurately account for tissue mechanics, contact angle, and applied force.

ARFI strain imaging, on the contrary, induces microscale displacement in tissues by using ARF (i.e., pushing pulse caused by sound energy) as an excitation method and tracks changes in mechanical properties as the tissue returns to baseline (Figure 2C) (43). This pulse, which is applied perpendicularly to the tissue surface, is typically short in duration (0.1–0.5 ms) and high-intensity (spatial peak pulse average = 1400 W/cm${}^{2}$, spatial peak temporal average = 0.7 W/cm${}^{2}$) (38). The resulting measurement of elasticity is displayed with brightness-mode (B-mode) ultrasound where darker regions correspond to stiffer tissue (Figure 2F).

A notable advantage of this technique is its ability to image beyond slip boundaries and stiff backgrounds (44). Slip boundaries refer to the interface between 2 tissues or anatomical structures that have different mechanical properties or stiffness (e.g., different types of tissue, or healthy tissue vs tumor). This interface is often challenging to visualize using conventional ultrasound imaging because the changes in echogenicity or reflectivity of these different types of tissues may not be prominent enough. However, ARFI strain imaging can detect differences in elasticity between tissues by measuring the mechanical wave reflection and refraction. While strain elastography is a simpler and more commonly used technique, ARFI strain imaging provides more reliable quantitative results due to its operator independence (38). This technique is highly useful in differentiating between benign and malignant breast and cervical masses, especially when used with B-mode imaging (43). Because malignant tumors are more firmly bound to background tissue compared with benign tissue, shear-strain distributions can effectively differentiate between the two.

Unlike strain elastography or SWE, ARFI has higher depth penetration and can image deeper tissue which is not reachable by manual compression. However, ARFI has limited spatial resolution and must be calibrated very carefully for adequate examination. Under ideal conditions, where tissue density and generation of shear waves are constant, the elasticity can be confidently calculated based on the shear wave velocity measurements. In clinical settings, however, the operator must take into account the environmental (i.e., physical, geometrical, and anatomical) factors that may modify the speed of shear wave propagation in the tissue of interest and lead to unreliable results (45).

### 3.2. Shear wave imaging

Shear wave imaging uses an applied stress (ARFI or mechanical vibration) to generate shear waves that propagate either parallel or perpendicular to the plane of excitation. This modality consists of 3 main approaches: 1D transient elastography (1D-TE), point shear wave elastography (pSWE), and 2D shear wave elastography (2D-SWE) (46). To better understand the relationship between elasticity and shear waves, Equation 1 correlates shear wave velocity to medium properties (47, 48):

(1) $$v=\sqrt{\frac{G}{\rho }},$$

where v is the shear wave velocity (m/s), G is the shear modulus (Pa) and ρ is the material density (${\text{kg/m}}^{3}$). The shear modulus is a quantitative value that describes a material’s resistance to shear deformation. As expected, higher shear wave velocities correspond to stiffer materials.

1D-TE is primarily used to assess the elasticity of deep tissue or organs, such as the liver. A mechanical device exerts a vibrating pressure on the surface to generate shear waves at a single point that propagate through the tissue (38). The shear waves parallel to the excitation source are then measured using amplitude-mode (A-mode) ultrasound to calculate the Young’s modulus. This technique is generally used for rapid assessment of tissue stiffness in a 1D representation at a specific location and because the acquisition time is short (typically less than 100 ms), measurements can be made on moving organs.

pSWE uses ARFI as an excitation method to generate displacement in a localized region of the tissue. By measuring the velocity of shear waves induced by ARFI at a single focal location, Young’s modulus of the medium can be determined to indicate material elasticity (49). The shear waves travel perpendicular to the plane of excitation. This modality does not require any special equipment (unlike 1D-TE, which needs a mechanically vibrating force) and can be performed with a conventional ultrasound machine and transducer. pSWE is favorable in deep tissue applications (e.g., liver) and because the shear waves are produced within the body using ARFI rather than superficially at the tissue surface with a vibrating device, the measurements are less affected by obesity (50).

In 2D-SWE, shear waves, which are generated by a force or a stress applied to a solid material, can be induced either mechanically by pressing down on the tissue or with an ultrasound transducer that generates ARF (51). By determining the time it takes for the wave to reach a focal point and the total distance traveled, the average velocity of propagation can be calculated and the resulting image color-codes the tissue based on the corresponding velocity (Figure 2E). 2D-SWE has several medical applications (e.g., liver fibrosis grading, tendon health monitoring) and provides an absolute tissue elasticity measurement (46).

Table 2 provides a comparison of the ultrasound elastography imaging technologies discussed: strain elastography, ARFI imaging, 2D-SWE, 1D-TE, and pSWE. Both strain and shear wave imaging are leveraged in the medical devices discussed in section 5, which focuses ultrasound systems that quantify tactile feedback and reviews their clinical applications.

**Table 2:** Modalities of ultrasound elastography.

Modality	Excitation	Advantages	Limitations
Strain elastography	Applied manual compression (38)	No additional specialized equipment required (40)	Qualitative measurements (39)
	Internal physiological mechanism (42)	Simple low-cost design (40)	Applied compression is operator-dependent (51)
		More commonly used (52)	High inter-observer variability (51)
coustic radiation force impulse (ARFI) imaging	Acoustic radiation force (43)	Image beyond slip boundaries (45)	Limited spatial resolution (45)
		Quantitative results (43)	
		Excitation is operator independent (43)	Requires careful calibration (45)
		Higher depth penetration (43)	
1D transient elastography (1D-TE)	Mechanical vibration generates shear waves (38)	Fast acquisition (38)	Ascites or increased thickness of adipose tissue (46)
		Widespread availability (49)	Fixed sampling area (single location) (49)
		Deep tissue elasticity assessment (38)	
Point shear wave elastography (pSWE)	ARFI generates shear waves in single focal zone (51)	Precise localization of tissue stiffness (51)	Fixed sampling area (small) (49)
		Can be performed in patients with ascites (46)	
		Visualization avoids vessels (46)	Increased thickness of adipose tissue (46)
2D shear wave elastography (2D-SWE)	ARFI generates shear waves in multiple focal zones (51)	Real-time monitoring of shear waves (51)	Signal artifacts at tissue-tumor interface (53)
		Greater field of view (51)	Increased thickness of adipose tissue (46)
		High reproducibility (54)	
		Not affected by ascites (46)	

## 4. Improvements with micromachined ultrasound transducers

Fundamental principles of ultrasound elasticity imaging can be applied to emerging technologies and algorithms to expand its uses in the medical field. Conventional piezoelectric transducers convert mechanical energy into electrical energy, with an active piezoelectric layer between 2 electrodes. The most common type of transducer for medical ultrasound imaging is developed with piezoceramics, which have high sensitivity but low efficiency for transmitting sound energy into tissue (55). The piezoelectric elements have been of significant interest as researchers strive to improve the performance of ultrasound transducers (i.e., sensitivity, resolution, power consumption) and thereby improve tactile feedback systems that rely on elasticity imaging. Since the advent of micro-electro-mechanical systems (MEMS) technology, there has been dedicated research efforts focused on overcoming these limitations. MEMS fabrication processes have enabled miniaturized and high-density electronic circuitry, resulting in devices with improved performance, along with reduced physical size and cost (56). Micromachined ultrasound transducers (MUTs), such as piezoelectric MUTs (PMUTs) and capacitive MUTs (CMUTs), are an advantageous alternative to traditional bulk ultrasound transducers developed with piezoceramics due to increased sensitivity from their high-density array architecture (57, 58).

### 4.1. Piezoelectric micromachined ultrasonic transducers

**Figure 3:**
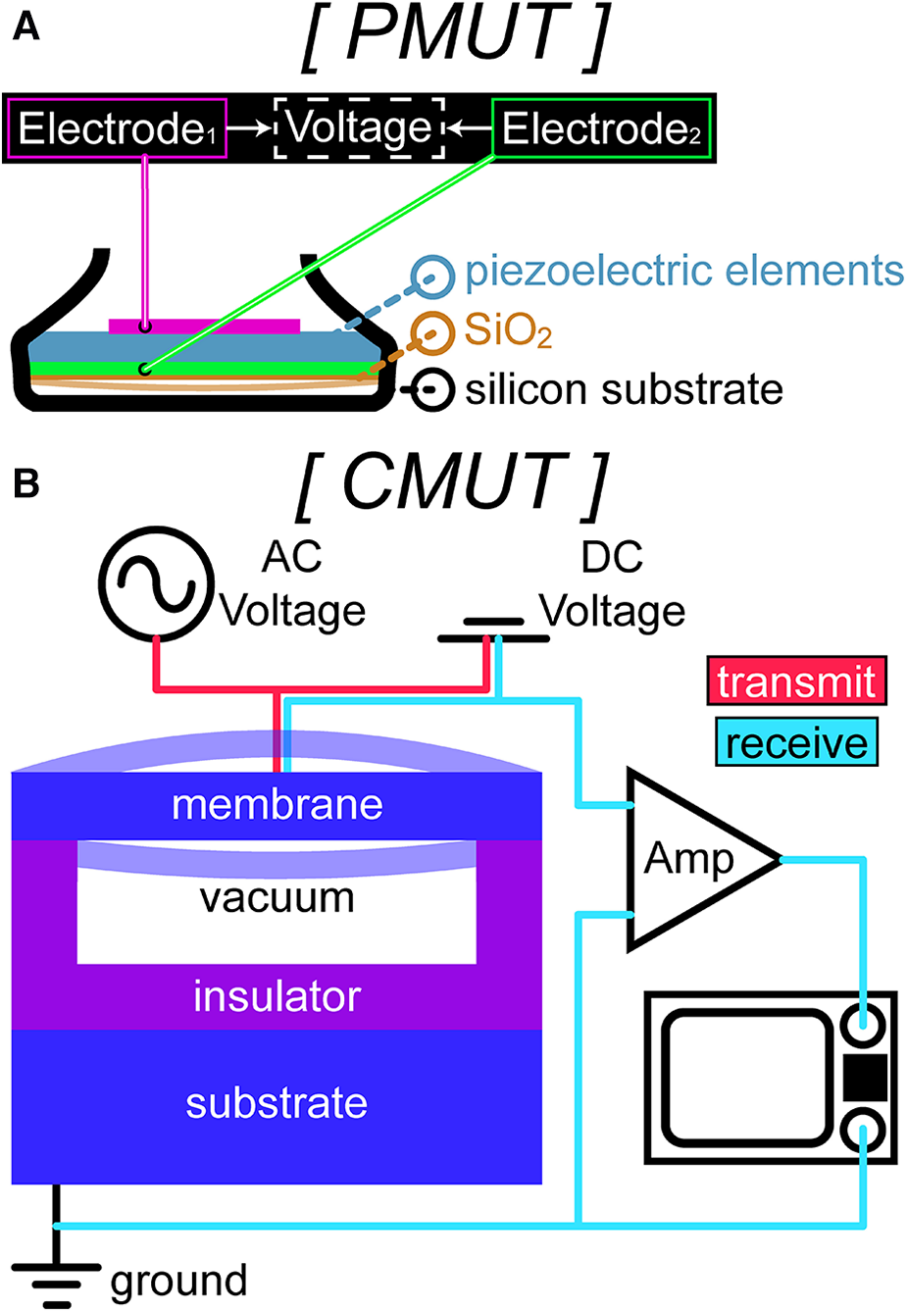
A sample schematic of (**A**) piezoelectric micromachined ultrasound transducer (PMUT) technology in a standard ultrasound probe. PMUTs have piezoelectric elements sandwiched between 2 electrodes and lie on top of a thin silicone dioxide membrane suspended over a silicon-based substrate. (**B**) Capacitive micromachined ultrasound transducer (CMUT) technology in transmit (red wiring) and in receive (cyan wiring). The bottom layer consists of a silicon substrate (bottom electrode) and the top layer is a membrane with a thin metal layer over it (top electrode) suspended over a narrow gap to prevent contact between the 2 electrodes for electrostatic actuation.

As opposed to bulk piezoelectric transducers, which require matching layers, PMUTs are devised on the principle of bending and vibrating a thin piezoelectric film (59). The piezoelectric layer is sandwiched between 2 electrodes and lies on top of a thin silicone membrane, and the bending of this layer from AC voltage excitation creates vibration at its resonant frequency (Figure 3A). PMUTs have a high piezoelectric coefficient, which corresponds to the high electrical energy generated per unit of applied mechanical force, leading to desirable performance metrics, high density, greater uniformity, and straightforward fabrication compared to other materials (60). Several factors play a role in the resonant frequency of the device, such as the size of elements, material selection, and bias voltage.

PMUT have been developed to achieve both superficial, high-resolution imaging and deep, low-resolution imaging (61, 62). Especially in diagnostic imaging, the resolution and depth of the image determine the sonographer’s ability to detect irregularities in tissue and vasculature. High-resolution images, created by high-frequency sound waves, suffer from greater signal attenuation, which ultimately results in smaller detection ranges. To expand the operating frequency of these transducers, beam-coupled PMUTs (BM-PMUTs) combine both low- and high-frequency elements into a single array. This design allows for 2 resonant frequencies, maintaining high sensitivity in both low- and high-frequency modes (3.5 and 18 MHz) so that deep and shallow regions can be visualized with a single hand-held probe (61, 62).

The high-density capabilities of PMUTs, such as real-time volumetric imaging, provide insight into tissue stiffness and occlusions in a reliable and refined manner. These functionalities, along with miniaturization, pave the future for advancements in tactile feedback system design.

### 4.2. Capacitive micromachined ultrasonic transducer

CMUTs have a similar design to PMUTs but without the piezoelectric film. While PMUTs operate using the piezoelectric effect, CMUTs operate by leveraging the electrostatic force between the top and bottom electrodes on the cell (63). In essence, a CMUT is a parallel plate capacitor, with a flexible top plate (membrane) and a fixed bottom plate (substrate) (Figure 3B) (59). CMUTs function by applying an AC voltage and a DC bias voltage simultaneously between the membrane and the substrate, which causes the membrane to move due to Coulomb forces, known as electrostatic actuation (64). The movement, as well as the vibration from the AC voltage excitation, generates ultrasonic waves (65).

CMUTs exhibit high sensitivity, increased resolution, and wide bandwidth (66). These arrays enable smaller inter-element spacing for improved spatial resolution and reduction of motion artifacts. Although cross-talk has presented several challenges in CMUT image quality, there is promising research in transducer fabrication, such as introducing a silica aerogel layer, for improving this issue (67). The easy integration of CMUTs with complementary metal-oxide semiconductors (CMOS) mitigates parasitic capacitance and improves signal-to-noise ratio. Additionally, because CMUTs have better acoustic impedance match to water and tissue compared to their piezoelectric counterparts, they achieve broader acoustic bandwidth and lower gain for biomedical applications.

### 4.3. Applications in elastography and considerations

MUTs not only have increased density of cells, but lower power consumption and acoustic impedance, allowing higher-quality ultrasound images (68, 69). High density fabrication leads to higher spatial resolution and more refined measurements, which is particularly important to visualize fine differences in elasticity within the tissue. These arrays also open opportunities for advanced beamforming techniques, primarily for improving image quality and reducing artifacts. Both PMUTs and CMUTs can be used for elastography, although they have different characteristics and advantages. CMUTs offer high sensitivity and are better suited for high-frequency ranges (>1 MHz), making them preferable for imaging small or shallow tissues, whereas PMUTs have high power-handling capabilities and perform better in low-frequency ranges (>40 kHz), which is ideal for imaging larger or deeper tissues (63, 68). Moreover, the increasing operating frequency range in PMUTs allows for improved signal-to-noise ratio, and therefore high quality imaging, at various tissue depths (61, 62, 70). MUTs demonstrate great potential in the field of tactile feedback due to their miniaturization, sensitivity, bandwidth, high density, and efficiency.

It is important to note, however, that the fabrication process for MUTs is complex and requires careful design and precise manufacturing. Because transducer development requires for the gap between elements to be less than half the wavelength in the medium to minimize side lobes from constructive interference of the ultrasonic wave, it is very difficult to dice a transducer that operates at high frequencies (62). Also, since PMUT arrays are traditionally fabricated using lead-based piezoceramics, biocompatible materials (e.g., Polyvinylidene fluoride (PVFL), poly-L-lactic acid (PLLA)) need further research and development to exhibit comparable cutting-edge performance (55). With these improvements, ultrasound for tactile visualization can make considerable leaps in diagnostic medicine.

## 5. Clinical tools using ultrasound to visualize and quantify tactile information

**Figure 4:**
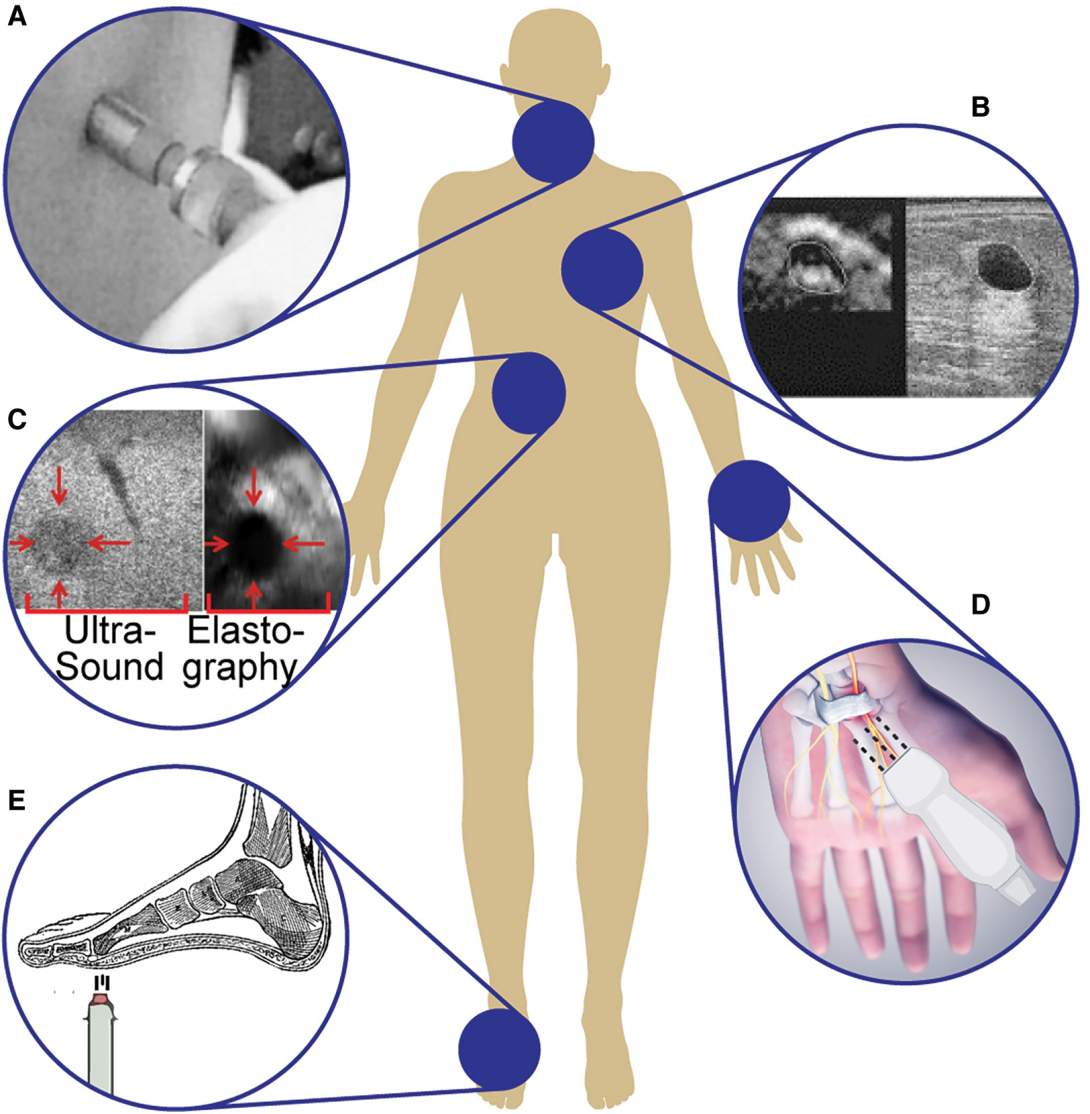
Examples of ultrasound systems for tactile information: (**A**) Hand-held ultrasound indentation system using acoustic radiation force in a pen-sized probe to assess neck tissue fibrosis (72). (**B**) Linear-array ultrasound transducers to image and differentiate breast lesions (73). (**C**) Automatic robotic-assisted palpation on the abdomen to generate a stiffness map to produce haptic feedback for remote or minimally invasive surgeries (74). (**D**) Ultrasound sensor to assess the transverse carpal ligament in carpal tunnel syndrome (75). (**E**) Differentiating diabetic and non-diabetic patients by studying the biomechanical properties of plantar soft tissues using an air-jet indentation system and ultrasound system (76).

This section delves into the current research in clinical systems that use ultrasound to visualize and quantify tactile information and its emerging use in medicine. We discuss the use of ultrasound elastography both in surgical contexts, where physical touch is limited, as well as for objective and quantitative assessments of tissue health for diagnostic purposes. Elastography demonstrates greater accuracy in tumor detection compared with manual palpation, as it can characterize deep tissue or nodules under layers of fat with increased reliability (71, 1). Here, we will highlight examples of robotic and hand-held systems that use ultrasound for clinical evaluations and their respective applications (Figure 4).

### 5.1. Neck tissue

With ARF, the user can employ a pen-size ultrasound probe to measure tissue displacement after applying stress to the tissue. By loading and unloading the probe onto the tissue, the difference in tissue elasticity can be computed using the continuously emitted ultrasound pulses. According to Zheng et al. (72), this technique provides sufficient accuracy and consistency for adequate assessment of neck tissue fibrosis (72). The hand-held ultrasound indentation system consists of a load cell (10 N strain gauge) to record the force response of the tissue and an ultrasound transducer (9 mm diameter, 5 MHz frequency) for imaging (Figure 4A). After applying pressure to palpate neck tissue, the Young’s modulus of the region of interest is calculated from the load-indentation response of the tissue. Unhealthy tissues (e.g., fibrosis) exhibited increased stiffness under loading, indicating that viscoelasticity parameters could discriminate soft tissue with varying degrees of fibrosis. Further research in identifying viscoelastic properties of soft materials (e.g., tissues) uses a low-cost dynamic indentation system to measure the shear modulus of a medium in response to applied pressure (77). A better understanding of tissue characteristics with quantitative measurements of viscoelasticity mitigates the inter-observer variations that often accompany manual palpation and operator-dependent imaging. The ability to identify variations in tissue elasticity with ARF is a result of greater independence from boundary conditions, decreased susceptibility to artifacts, and a reduced rate of decay of strain signal-to-noise ratio compared with conventional elastography (78). The effectiveness of ARF is highly dependent on the skill and experience of the operator and is limited by the penetration depth of the ultrasound waves.

### 5.2. Breast lesions

Additionally, some ultrasound systems use linear array transducers to image and differentiate breast lesions, using real-time visual feedback of tissue elastic properties to guide positioning and compression (Figure 4B) (73). This system provides high contrast-to-noise ratios in the resulting freehand elasticity maps which are displayed with the corresponding B-mode image (52). To assess its sensitivity to different medical conditions, the transducer was tested on 3 commonly observed breast lesions (i.e., fibroadenomas, cysts, and carcinomas), and each type resulted in significantly different strain-image sequences. The lesion boundary determinations were reproducible with this system, and lesion dimensions (both height and width) were measured with high confidence (52). To further the clinical applications of ultrasound for breast tumor localization, various research groups have applied deep learning architectures to automate and improve the diagnostic utility of B-mode ultrasound (79, 80). It has been shown that deep learning models are able to distinguish between benign and malignant breast masses on ultrasound SWE with equal or greater accuracy compared to trained radiologists (81). These computational techniques have significant implications for ultrasound elasticity imaging, especially in situations where image quality may suffer (e.g., hand-held probes with limitations on element density).

### 5.3. Abdomen

An advantageous application of elastography to obtain tactile information is in robotic surgeries, where surgeons have limited or no physical access to the region of interest. Several research efforts are underway to integrate elastography in robotic systems in an effort to gain the same information from manual palpation in these settings. B-mode supplemented with laparoscopic elastography allows clinicians to differentiate between lesions with various elasticity and identify the lesion boundary with increased confidence compared to using a single modality alone (82, 35). The image is generated by applying varying levels of strain to the tissue at periodic time intervals. The ultrasound probe simultaneously palpates and images the tissue, inducing strain under different amounts of applied pressure. These images are then used to create a displacement map to understand the relative stiffness of the underlying tissue. Because the robot-assisted system has precise control of probing depth and contact angle, the quality of the strain elastography images are optimized and repeatable compared to manual compression.

To increase the ease and accuracy of this method, automatic robot-assisted elastography was developed to allow for teleoperative control of the ultrasound probe and provide haptic feedback to the clinician to complement the resulting strain elastography image (Figure 4C) (74). The continuous palpation motion with an ultrasound probe in this robotic system enables real-time estimations of the elastic parameters of tissue, allowing for increased accuracy in tumor detection and localization. These elasticity parameters are rendered with haptic force feedback during real-time tissue examinations to artificially restore manual palpation. The robot-assisted system generates force from the estimated elastograms produced by the automatic palpation movement of the robotic ultrasound probe and renders it through the haptic device (74). The force is calculated based on the general principles of elastic Hooke’s law (Equation 2),

(2) F=−A∗E∗S,

where F is the reactive force generated by an elastic material, A is the cross-sectional area of the region of interest (i.e., where the stress is applied), E is the Young’s modulus of the material, and S is the observed strain. This system has been evaluated with an abdominal phantom, exhibiting promising results for clinical use; however, additional developmental work is needed to validate its functionality in various types of tissue (74).

When designing medical robotics for ultrasound imaging and tissue elasticity assessment, there are several considerations to address before commercialization and clinical practice. The system must be rigorously and robustly tested for patient safety as there are potential dangers of clamping, squeezing, and applying uncomfortable pressure. Additionally, integration of remote control and raw ultrasound data access requires development of open platforms and collaboration, as these processes in commercial systems are proprietary (83). For these reasons, there are currently only commercially available teleoperated ultrasound systems: MGIUS-R3 (MGI Tech Co.) system (San Jose, California, USA) and MELODY (AdEchoTech) system (Mississauga, Ontario, CA) (84, 85). While patients and examiners accept telesonography for improving access to care, advanced solutions to image quality, autonomy of image acquisition, and robotic navigation are necessary to facilitate commercialization and to eventually reach a fully independent platform (86). Even though autonomous operation has not yet been achieved, there are several research efforts working to accomplish this goal, discussed in (83). Artificial intelligence is at the forefront of these efforts, playing a significant role in robotic path planning. It has several uses, ranging from creating a patient-specific body atlas by segmenting organs based on MRI data to compensating for motion and deformation noise commonly associated with ultrasound for image analysis.

These ultrasound-based robotic systems can also be improved with the use of tactile sensing (i.e., resistive, capacitive, piezoelectric, optical), which has been demonstrated in the context of minimally invasive surgeries (87). The integration of these sensor arrays with ultrasound introduces a multi-modal clinical tool to remotely detect pressure and to measure the underlying stiffness of an object to achieve more reliable tissue assessments. The low-cost disposable sensors (49×10×2.5 mm${}^{3}$, 90 sensing elements, 30 Hz update rate) combined with the linear-array transducer (128 elements, 4–9 MHz operating frequency range) have been shown to achieve higher tumor localization accuracy compared to evaluations with ultrasound alone (87).

Developing research in liver elastography using CMUTs is an interesting alternative to overcoming the limitations of traditional approaches like 1D-TE. Due to signal degradation from subcutaneous fat or large exploration depth, a transducer with a broader frequency range would be beneficial. This would allow for precise adjustment of the operating frequency to minimize tissue attenuation. CMUTs are particularly beneficial in this regard, as they typically have a frequency bandwidth of 110%. Certon et al. (88) reported that a fabricated single-cell CMUT connected to a FibroScan device (Echosens, Paris, France) produced comparable shear wave maps to one from a counterpart piezoelectric (PZT) probe (88). In this study, a CMUT cell (8mm×8mm) was designed with a surface micromachining process to have similar acoustic characteristics to the PZT-single element (8 mm diameter) used for 1D-TE. Both of these probes were used on an acoustic phantom (8.5 kPa stiffness) at a frequency of 2.5 and 5 MHz and the measured shear wave speed for the CMUT and PZT probes were 1.65 and 1.68 m/s, respectively. While many improvements need to be made to the CMUT design, such as increasing the element surface ratio, mechanical focusing, and developing an electronic front-end, there is promise for CMUT-based elastography applications. Moreover, in (89), a 2D CMUT array (center frequency 7.5 mm) was developed for high intensity focused ultrasound (HIFU) and imaging. In HIFU mode, the transducer emits a short high intensity ultrasound wave (shear wave) to the target region in the liver to induce a deformation in the tissue. This applied strain is then measured in elastography mode, which captures an image and calculates shear wave speed of the deformed tissue. While this device still needs to be rigorously tested and evaluated for performance and safety, it is working towards establishing a non-invasive, accurate, and less expensive alternative to other diagnostic methods (e.g., liver biopsy, blood test, MRI, CT) (89).

### 5.4. Hand tendons

A tissue ultrasound palpation sensor can assess the transverse carpal ligament by examining the thickness and stiffness of the transverse ligament in carpal tunnel syndrome (Figure 4D) (75, 90). This sensor is connected to a personal computer via a universal serial bus (USB) to provide real-time signal and indentation force information and an improved user interface. The finger-sized probe, consisting of an ultrasound transducer (5 MHz operating frequency) and load cell, pushes against soft tissue to measure both the thickness and elastic profile of the region of interest. The compression force (20 N) is applied with a cylindrical ultrasound indenter (9 mm diameter), and the deformation within the wrist during indentation is calculated based on the speed of sound and time-of-flight of the ultrasound signal. With motion-mode (M-mode) ultrasound, his system distinguishes between the different layers of tissue (e.g., soft tissue, carpal tunnel) within the wrist, rather than solely providing an overall measurement of tissue thickness and elasticity. Chen et al. (91) demonstrated that 2D shear wave velocity images of hand tendons could be mapped for healthy and injured tendons using high-frequency ultrasound elastography (40 MHz) and a handheld vibration system to continuously vibrate and measure shear wave speed synchronously (91). This system overcame the previous barrier of high-resolution shear wave imaging, with a spatial resolution of 147 μm for a more accurate assessment of small tendon stiffness in hands.

Since tendons change in rigidity depending on their health, elastography is a promising tool to monitor the severity of hand injuries and assess their healing during rehabilitation. Due to the small size of tendons in the hand (on the scale of a few millimeters), high-resolution imaging with high-frequency ultrasound SWE and a continuous vibration method is used to measure the shear wave velocity (92). This technique employed a 40-MHz ultrasound array transducer in a hand-held probe to characterize hand tissue and was successfully able to differentiate between healthy and injured tendons.

### 5.5. Plantar soft tissue

The handheld capabilities of tissue ultrasound palpation sensors have also been applied to the study of biomechanical properties of plantar soft tissues to determine characteristic differences between diabetic and non-diabetic subjects (Figure 4E) (76). This study used an ultrasound transducer (10 MHz, 3 mm diameter) and a 10 N load cell to record the force during indentation to complete an offline analysis of the stiffness of tissues. The strain and applied stress were determined based on ultrasound time of flight, using a pen-style probe and a single-channel pulser–receiver with an analog-to-digital converter. The transducer was covered with a deformable elastomer tip, and the axial deformation during palpation was then used to calculate the applied force. The corresponding stress and strain were calculated in real-time in pulse-echo mode, where the transducer emits short pulses and measures the time to receive the reflection (93).

## 6. Discussion and conclusion

Palpation has been long used by clinicians for diagnostic purposes. However, it presents many limitations and requires supplemental technology for quantitative information. Although many studies have discussed tactile sensors for numerically identifying the tissue’s feedback response, this review focuses on the potential of ultrasound to detect and image the stiffness of both superficial and deep tissue.

Recent advancements that allow ultrasound to be integrated into convenient, cost-effective, pen-sized probes have facilitated the convergence of ultrasound elasticity imaging with palpation. Furthermore, ultrasound can be used in situations where palpation is not possible, such as remote or minimally invasive surgeries. The small size of the incision, while necessary for shorter healing times, prevents surgeons from palpating organs and eliminates tactile feedback. This physical assessment can be restored visually and quantitatively via ultrasound. Along with presurgical diagnosis and surgical navigation, ultrasound is an excellent candidate for applications in needle and catheter guidance, with increased success rate of cannulation particularly in patients with difficult intravenous access (94–96). Current state-of-the-art hand-held ultrasound probes use computational approaches to produce high-resolution and easily interpretable images, but lack the elasticity imaging that is necessary for real-time quantitative assessment of tissue health (97).

Compared to other imaging modalities, such as radiography, MRI and CT, ultrasound is safer, more affordable, and portable (11). Because ultrasound images are captured in real-time, they can show movement of internal organs and blood flow, and visualization of these features is not possible with the aforementioned imaging techniques. Additionally, there is no radiation exposure or health detriments associated with sonography, unlike with radiography and CT. Modalities like MRI, which require significant space to generate magnetic fields for imaging, demand extreme stillness from the patient for an extended period, which is not only uncomfortable and slow, but also is unfeasible in some cases (e.g., in the pediatric setting).

Although ultrasound introduces numerous advantages, certain limitations need to be addressed, including probe size, requirements for an acoustic coupling interface (e.g., gel), and user proficiency needed to adeptly obtain and interpret the ultrasound images. Furthermore, because ultrasound cannot image bone due to high attenuation and distortion of the acoustic wave, noninvasive imaging of organs fully encompassed in bone, such as the brain or spinal cord, poses a challenge. Presently, surgical procedures that remove a portion of the skull (i.e., craniotomy) or the lamina surrounding the spinal cord (i.e., laminectomy) are required to gain access to real-time imaging. Techniques described by Lu et al. (98) investigate the use of an anisotropic, acoustic complementary metamaterial to restore acoustic fields that are distorted by bone and similar impenetrable materials (98). This material has been demonstrated to acoustically cancel out the effects of aberrating layers and noninvasively enhance sound transmission.

Future works can be centered on machine learning approaches to provide automatic localization and classification of tumors in ultrasound images based on tissue elasticity. This direction can further lead to automated clinical tools for accurate computer-aided diagnosis and improved patient outcomes. There are several research efforts on advanced beamforming (99, 100) and deep learning (101, 102) to address some of the current drawbacks of ultrasound imaging and elastography. Shadowing, which is an artifact at interfaces with high acoustic impedance mismatch (e.g., soft tissue and air), can inhibit the operator’s ability to identify abnormalities because of overwhelming ultrasound wave absorption or reflection. This results in signal loss and dark shadows that obscure the region of interest. Another limitation is reverberation, in which ultrasound beams are trapped in between 2 strong reflectors, which significantly degrades image quality and accuracy. We commonly observe this in lung scans, where the ultrasound beam can be reflected multiple times between the pleural surface and the skin-transducer interface (103). Finally, acoustic cluttering, as the name suggests, results in noise and speckling in the image, which detracts from focusing on a region of interest (104). These current imaging impediments need to be addressed with image and signal processing techniques to assist clinicians and further improve the benefits of using ultrasound with palpation.

Integration with tactile sensors for a multi-modal approach can also help mitigate the current drawbacks of these ultrasound-based systems while retaining the added benefit of visualization and high sensing depth (87). The clinical accessibility of ultrasound elastography will only continue to improve as breakthroughs in wearable technology are made, allowing for serial assessment of tissues to aid with early detection and continuous supervision of pathophysiological conditions (105). Hu et al. (106) has demonstrated a stretchable ultrasonic array for measuring tissue elasticity up to 4 cm beneath the skin (106). This flexible array conforms to skin and maps 3D distributions of the Young’s modulus to track the evolution of lesions at a spatial resolution of 0.5 mm. The accelerating field of flexible ultrasonics combined with artificial intelligence for automatic classification of lesions and prediction of the trajectory of diseases is facilitating a future for accessible and preventative medicine. Increased reliability and sensitivity in state-of-the-art ultrasound systems compared with traditional methods of localizing unhealthy tissue can lead to the treatment of diseases before they progress to a more insidious state.

## Author contributions

AK, AM, and NVT devised the review topic and scope, and AM and NVT provided their expertise in ultrasound and tactile feedback sensors to revise manuscript drafts. AK conducted the literature search and wrote the manuscript. KMKL designed Figures 2D–F, helped with review organization, and provided feedback on the manuscript drafts. MJK designed Figures 2A–C and 3 and provided feedback on the manuscript drafts. SS designed Figures 1 and 4 and provided feedback on the manuscript drafts.

## Funding

AM acknowledges funding support from the National Science Foundation (NSF) STTR Phase 1 Award (#: 1938939), Defense Advanced Research Projects Agency (DARPA) Award (#: N660012024075), and Johns Hopkins Institute for Clinical and Translational Research (ICTR)’s Clinical Research Scholars Program (KL2), administered by the National Institutes of Health (NIH) National Center for Advancing Translational Sciences (NCATS).

## Conflict of interest

The authors declare that the research was conducted in the absence of any commercial or financial relationships that could be construed as a potential conflict of interest.
